# Only a small proportion of patients with first episode psychosis come via prodromal services: a retrospective survey of a large UK mental health programme

**DOI:** 10.1186/s12888-017-1468-y

**Published:** 2017-08-25

**Authors:** Olesya Ajnakina, Craig Morgan, Charlotte Gayer-Anderson, Sherifat Oduola, François Bourque, Sally Bramley, Jessica Williamson, James H. MacCabe, Paola Dazzan, Robin M. Murray, Anthony S. David

**Affiliations:** 1 Department of Psychosis Studies, Institute of Psychiatry, Psychology & Neuroscience, King’s College London, 16 De Crespigny Park, London, SE5 8AF UK; 2 Society and Mental Health Research Group, Health Service and Population Research Department, Institute of Psychiatry, Psychology & Neuroscience, King’s College London, De Crespigny Park, London, SE5 8AF UK; 3 NIHR Biomedical Research Centre, David Goldberg Building, Institute of Psychiatry, Psychology & Neuroscience, Kings College London, De Crespigny Park, London, SE5 8AF UK; 4 Guy’s, King’s and St Thomas’ School of Medical Education, King’s College London, London, UK; 5 Violence Prevention Research Unit Queen Mary University of London & East London NHS Foundation Trust, Garrod Building, Turner Street, London, E1 2AD UK; 6 National Institute for Health Research (NIHR) Mental Health Biomedical Research Centre at South London and Maudsley NHS Foundation Trust and King’s College London, London, UK

**Keywords:** First episode psychosis, At risk mental state, Pathways to care, Retrospective, Transition to psychosis, Prodromal services

## Abstract

**Background:**

Little is known about patients with a first episode of psychosis (FEP) who had first presented to prodromal services with an “at risk mental state” (ARMS) before making the transition to psychosis. We set out to identify the proportion of patients with a FEP who had first presented to prodromal services in the ARMS state, and to compare these FEP patients with FEP patients who did not have prior contact with prodromal services.

**Methods:**

In this study information on 338 patients aged ≤37 years who presented to mental health services between 2010 and 2012 with a FEP was examined. The data on pathways to care, clinical and socio-demographic characteristics were extracted from the Biomedical Research Council Case Register for the South London and Maudsley NHS Trust.

**Results:**

Over 2 years, 14 (4.1% of *n* = 338) young adults presented with FEP and had been seen previously by the prodromal services. These ARMS patients were more likely to enter their pathway to psychiatric care via referral from General Practice, be born in the UK and to have had an insidious mode of illness onset than FEP patients without prior contact with the prodromal services.

**Conclusions:**

In the current pathways to care configuration, prodromal services are likely to prevent only a few at-risk individuals from transitioning to psychosis even if effective preventative treatments become available.

**Electronic supplementary material:**

The online version of this article (doi:10.1186/s12888-017-1468-y) contains supplementary material, which is available to authorized users.

## Background

Psychotic disorders and schizophrenia in particular historically are progressive deteriorating conditions. A long duration of untreated psychosis (DUP) is believed to be an important contributing factors to the severity of the illness and subsequent poorer outcomes [1]. This has added to the focus on specialist early intervention (EI) services for first episode psychosis (FEP) [2, 3] the aim of which is to reduce treatment delay, and thus to increase chances for recovery and to improve overall prognosis of psychosis [1]. Even though some studies have suggested that the benefits of EI services may be negligible [4], the British EI services are designed to intervene during the first three years after the illness onset [5] - a period that is perceived to be crucial in shaping the prognosis of the illness [6] - by means of administering low doses of antipsychotics, family involvement, education and support [5]. There is also evidence showing that EI indeed reduces DUP once patients entered the programme [7]. In the UK pathways to care for FEP are diverse. The most widely accepted route of access to mental health services is through general practitioners (GPs), who then refer the patients to specialist psychiatric services [8]. However, more often than not, other non-healthcare agencies such as police and the criminal justice system serve as the first point of contact for individuals in crisis with their first onset of psychosis [8].

The FEP is often (but not always) preceded by a phase termed the At Risk Mental State (ARMS) [9], which is characterised by either ‘attenuated’ psychotic symptoms, or full blown psychotic symptoms that are brief and self-limiting. The ARMS period may also manifest as a significant decrease in functioning in the context of a genetic risk for schizophrenia or subtle subjective disturbances of cognitive processes, changes in thinking, perception, mood, affect and behaviour [10, 11]. Up to 30–45% of those with the ARMS are claimed to develop a psychotic disorder in the following 24 months [12]; though with some reporting transition rates 11–17% within a 2-year period [13, 14]. Identification of the ARMS individuals therefore provides a unique opportunity to attempt to prevent such individuals transitioning to clinical psychosis [1, 9, 15]. This recognition has provoked the development of prodromal services that provide comprehensive care to young individuals meeting criteria for the ARMS [9, 11, 16, 17]. Having established close links with primary care providers and non-health related community services, such as schools, counsellors, and emergency and criminal justice agencies, prodromal services provide an accessible and acceptable service for help-seeking young people who are at risk of psychosis [9, 18].

Surprisingly, to our knowledge there has been no published report from anywhere in the world on the proportion of individuals who present to psychiatric services with FEP and who have previously accessed mental health care via prodromal services. Compared to the standard EI services, access to the prodromal services requires patients to demonstrate active help-seeking [9, 18], a requirement that may render individuals who attend such services unrepresentative of the overall at risk population. Moreover, knowledge of pathways to care for those at high risk of psychosis remains incomplete [19] as does how such pathways differ from those used by the remainder of patients with a FEP [20]. A better understanding of how different groups interact with healthcare systems may enable such systems to more effectively target interventions aimed at preventing transition to clinical psychosis in those with an ARMS [20].

The aims of the present study are two-fold. First, we set out to identify the proportion of patients with FEP who had first presented to the local prodromal services in the ARMS state before making the transition to FEP. Second, we sought to compare clinical and socio-demographic characteristics and differences in pathways to care between such FEP patients and those FEP patients presenting directly to standard first episode services, all within a tightly defined catchment area served by the South London and Maudsley NHS Foundation Trust (SLaM). We hypothesised that the interval between the first onset of symptoms and initiation of treatment would be shorter in those attending the prodromal services compared with those patients referred to conventional services. Furthermore, although a number of earlier studies have shown that a number of patients who are referred to the prodromal services are already experiencing a first episode of psychosis at the time of the contact [9, 21], it is not known whether this subgroup of patients differs from FEP patients who present to generic services, perhaps because of factors related to help-seeking. We additionally investigated this question by comparing the those individuals who were referred to prodromal services with a suspected ARMS but in fact were already experiencing a full clinical first psychotic episode with those FEP patients who did not have prior contact with the prodromal services before developing a FEP in terms of socio-demographic characteristics, duration of untreated psychosis, clinical presentation and pathways to care.

## Methods

### Sample

Patients in this study were identified as part of the European Union Gene-Environment Interactions (EU-GEI) study and presented to mental health services in the South London and Maudsley NHS Foundation (SLaM) Trust between 1 May 2010 to 30 April 2012 with a FEP (International Classification of Diseases [ICD-10] codes F20-F29 and F30-F33) (World Health Organisation [WHO], 1992) validated by administration of the Schedules for Clinical Assessment in Neuropsychiatry [22]. The age limit in this study was 37 years old and was chosen to be consistent with the age limit for patients attending the ARMS clinic (35 years) plus 2 years to allow them to present with a FEP. The patients were included in the study if they were current residents of Lambeth (population 303,086) or Southwark (population 288,238) [23] boroughs served by the Trust. Exclusion criteria were: 1) evidence of psychotic symptoms precipitated by an organic cause; 2) transient psychotic symptoms resulting from acute intoxication as defined by ICD-10; and 3) head injury causing clinically significant loss of consciousness. No specific screening criteria were used.

The FEP patients were further subdivided into 3 groups: 1) FEP patients who had first presented to the prodromal services with the ARMS and who, by definition, subsequently transitioned to FEP (i.e., *PROD* group); 2) FEP patients without prior contact with the prodromal services before their first contact with the mental health services for FEP (i.e., *FEP-C* (control) group); and 3) patients who were found to be already experiencing their first psychotic episode at the time of first contact with the prodromal services (i.e., *FEP-P* (psychosis) group).

### Data source

The patients for this study were identified from electronic records obtained from the SLaM Biomedical Research Centre (BRC) Case Register Interactive Search tool (CRIS). The SLaM, whose case records provide the source data for the SLaM BRC Case Register, is the largest provider of secondary mental healthcare in Europe, covering a socially diverse region of 1.2 million residents in South East London [24]. Within the UK National Health Service context, effectively all secondary mental health services are managed and provided by single, geographically-defined mental health trusts (such as SLaM).

CRIS was developed in 2008 and consists of a series of data-processing algorithms which effectively anonymise [25], structure and extract all electronic health records, including correspondence, discharge letters and events, reported by treating clinicians throughout patients’ journeys through the Trust services [24–26]. Using the CRIS system, we identified all patients who came in contact with the SLaM for a FEP over 2 year period. Where there was ambiguity about the FEP status of a patient, a consensus decision was made by members of the research team; this always included those with long-standing expertise in first episode psychosis (C.M.). The CRIS was further utilised to extract information on socio-demographic characteristics, clinical presentation and pathways to care for all identified patients. Additionally, we used CRIS to identify those patients from our cohort of FEP cases who were referred to the Outreach and Support in South London Service (OASIS) [9, 17] services and who, having met criteria for the ARMS (using the Comprehensive Assessment of the At Risk Mental State (CAARMS) [27]), were accepted for treatment prior to making the transition to FEP.

A comprehensive description of these EI services and how they operate is provided elsewhere [9, 17]. Briefly, OASIS is a specialised community mental health service for people aged 14–35 years old with the ARMS for psychosis. It is one of the largest and most well established prodromal services in the world and is well integrated with the first-episode services in all main South London boroughs. OASIS accepts referrals via telephone, mail and fax, which can be made by individuals themselves, their friends, relatives, mental or non-mental health professionals as well as non-health agencies including educational establishments, community services and churches. OASIS team responds promptly to all referrals and conducts the first assessment within the first week of the referral being made and provides a 2-year follow-up to those with ARMS [18].

### Assessments

#### Socio-demographic characteristics

The Medical Research Council (MRC) Socio-demographic Schedule (modified version) was utilised to extract data on socio-demographic characteristics and cannabis use [28]. Ethnicity was self-ascribed and recorded on the clinical notes by the treating clinicians and was further classified using the 18 categories employed by the 2011 UK Census (http://www.ons.gov.uk/census/2011census). Due to relatively small numbers of patients in some of our groups, we combined the ethnic categories into three broad ethnic groups: white (all white groups), black (all black groups), and other (encompassing Asian, mixed and other ethnicities).

#### Clinical presentation

Duration of untreated psychosis (DUP) was defined as the time between the date of an appearance of first symptoms of psychosis and date of start of first treatment with antipsychotic medications [29]. Age at first contact was defined as the age at which a patient was in contact with mental health services for the first time [30]. Similar to previous studies [31, 32], mode of onset of psychotic symptoms was operationalised using definitions developed for the World Health Organisation International Pilot Study of Schizophrenia, and was categorised into three groups: 1) *acute* (psychotic symptoms appeared within hours, 1 week or 1 month of first noticeable behavioural change); 2) *gradual* (psychotic symptoms appeared within period of 1 to 6 months of first noticeable behavioural change); and 3) *insidious* (psychotic symptoms appeared incrementally over a period of 6 months or greater since first noticeable behavioural change).

#### Pathways to care

The four most commonly used pathways were examined: 1) general practitioner (GP); 2) emergency medical services (primarily hospital accident and emergency departments, and walk-in centres); 3) criminal justice agencies (police, prison or probation services and courts); and 4) health workers (social support workers, nurses or other mental health workers).

### Statistical analysis

The distributions of socio-demographic characteristics, clinical presentation and pathways to care were explored with frequencies, percentages, mean and standard deviation, median and inter-quartile range (IQR). The comparisons between the groups were made using *x* ^2^ test or *Fisher*’s exact test when expected cell counts were less than 5 [33] for categorical data and t-tests for continuous data. DUP was heavily skewed and was consequently log-transformed to allow parametric analyses. DUP for each group of patients is presented in the original scale, while the analyses were conducted using the logarithmic-transformed values. To test the differences in clinical presentation and pathways to care between the groups of patients on their first contact with the services independent of confounding factors we employed exact logistic regression (ELR), which is an ideal and methodologically logical alternative approach to the maximum unconditional likelihood method when sample sizes are small or the data are sparse [34]. As it has been shown that in order to maintain the validity of the models the ratio of the number of patients suffering endpoints to the number of potential predictors should be at least 10:1 [35, 36], we adjusted our ELR models for one confounding variable; that is age at first contact with mental health services. All analyses were conducted in STATA release 14 (STATACorp, USA).

## Results

### Sample characteristics

**Fig. 1:**
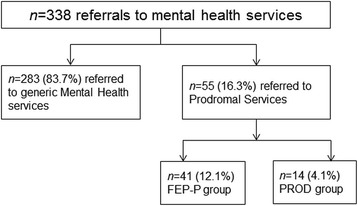
Depicts the information on identification of patients with a FEP who had first presented to mental health services in the ARMS phase of psychosis and who, by definition, subsequently transitioned to a psychotic disorder, and FEP patients who did not have a prior contact with prodromal services

The information on the groups is illustrated in the Fig. 1. Between 2010 and 2012, there were 338 referrals aged ≤37 years old for first episode psychosis within catchment boroughs served by the SLaM Trust. Of these, 283 (83.7%) were referred to conventional mental health services for FEP without prior contact with the prodromal services (i.e., FEP-C group). Overall, 55 (16.3% of 338) FEP patients had been in contact with the prodromal services. Of these, 14 (25.5% of 55 and 4.1% of 338 FEP cases) met criteria for the ARMS and subsequently transitioned to FEP (i.e., PROD group); the remaining 41 (74.5% of 55 and 12.1% of 338) already met criteria for a clinical psychotic disorder at the time of first contact with the prodromal services (i.e., FEP-P group).

### Socio-demographic characteristics: FEP-C vs PROD groups

Comparisons in socio-demographic characteristics between the FEP-C and PROD groups at the time of first contact with mental health services are presented in Table 1. At the time of first contact with mental health services, the FEP-C group was older (mean = 27.9 years, S.D. = 5.5) than the PROD group (mean = 24.2 years, S.D. = 6.0) (*t*(295) = 2.46, *P* = 0.01). A higher proportion of the PROD group (*n* = 10/14, 83.3%) was born in the UK compared with the FEP-C group (*n* = 124/234, 47%) (*x* ^*2*^ = 6.14, *P* = 0.01).

**Table 1:** Comparisons in socio-demographic variables between FEP-C (control) and PROD (prodrome) groups

Socio-demographic characteristics	FEP-C (*n* = 283; 83.7%)	PROD (*n* = 14; 4.1%)	Statistics		
	Mean (S.D.)/n(%)	Mean (S.D.)/n(%)	Test statistics	df	*P*-value
Age, y	27.9 (5.5)	24.2 (6.0)	*t* = 2.46	295	0.01
Range	18–37	15–34			
Gender			chi^2^ = 0.36	1	0.60^a^
Female	124 (43.8)	5 (35.7)			
Male	159 (56.2)	9 (64.3)			
Ethnicity			chi^2^ = 2.65	2	0.29^a^
White	98 (35.1)	7 (53.8)			
Black	126 (45.2)	3 (23.1)			
Other	55 (19.7)	3 (23.1)			
Country of birth			chi^2^ = 6.14	1	0.02^b^
UK	124 (46.8)	10 (83.3)			
Not in the UK	141 (53.2)	2 (16.7)			
Education			chi^2^ = 1.30	1	0.21^b^
School	111 (55.2)	8 (72.7)			
A-Level, or above	90 (44.8)	3 (27.3)			
Employment status			chi^2^ = 1.24	1	0.27^a^
Unemployed	172 (64.7)	7 (50.0)			
Employed	94 (35.3)	7 (50.0)			
Marital status			chi^2^ = 2.98	1	0.10^a^
Not in stable relationship	207 (75.3)	7 (53.9)			
Married/stable relationship	68 (24.7)	6 (46.1)			
Living arrangements			chi^2^ = 4.08	2	0.15^b^
Alone	74 (27.0)	1 (7.1)			
Partner/family	125 (45.6)	10 (71.4)			
No stable accommodation	75 (27.4)	3 (21.4)			
Cannabis use			chi^2^ = 1.86	1	0.26^b^
No	118 (50.2)	4 (30.8)			
Yes	117 (49.8)	9 (69.2)			

*ARMS* At Risk Mental State, *IQR* inter-quantile range, *S.D.* standard deviation, *df* degree of freedom, *y* years, *FEP-C (control) group* FEP patients who present to mental health services for FEP without prior contact with the prodromal services, *PROD (prodrome) group* FEP patients who had first presented to the prodromal services with the ARMS and who, by definition, subsequently transitioned to FEP

^a^chi-square test

^b^Fisher’s exact test

### Clinical presentation and pathways to care: FEP-C vs PROD groups

Comparisons in clinical presentation and pathways to care between the FEP-C and PROD groups at the time of first contact with mental health services are presented in Table 2. DUP was highly skewed; the median length of DUP was substantially longer in the FEP-C group (median = 86 day, IQR = 13–368) compared with the prodromal group (median = 19 day, IQR = 6–40), albeit the difference was not statistically significant at the conventional level of *P* < 0.05 (*t*(219) = 1.35, *P* = 0.18). Further, 73% (*n* = 8/11) of the PROD group and 36% (*n* = 100/277) of the FEP-C group had an insidious mode onset. Indeed, the PROD group was more likely to have an insidious mode of psychosis symptoms onset compared to those patients with FEP who did not have prior contact with prodromal services (OR = 5.17, 95% CI = 1.18–31.51). Moreover, the pathways to care were varied among the groups: 45% (*n* = 124/274) of the FEP-C group made first contact with mental health services via emergency medical services and 18% (*n* = 49/274) of this group were referred by the criminal justice system. By contrast, 77% (*n* = 10/13) of the PROD group entered their pathway to care via referral from GPs or other health professional. FEP patients who had first presented to the prodromal services with the ARMS and who subsequently transitioned to FEP were less likely to make their first contact with mental health services via emergency services compared to those FEP patients who did not have prior contact with prodromal services (OR = 0.19, 95% CI = 0.02–0.91).

**Table 2:** Comparisons in clinical presentation characteristics and pathways to care between FEP-C (control) and PROD (prodrome) groups

Clinical presentation and pathways to care	FEP-C (*n* = 283; 83.7%)	PROD (*n* = 14; 4.1%)	Unadjusted OR (95% CI)	Adjusted^a^ OR (95% CI)
	Median (IQR)/n(%)	Median (IQR)/n(%)		
DUP, d	86 (13–368)	19 (6–40)	0.99 (0.96–1.00)	0.99 (0.96–1.00)
Source of referral				
General Practitioner	67 (24.4)	6 (46.2)	2.64 (0.71–9.53)	2.82 (0.75–10.34)
Emergency services	124 (45.3)	2 (15.4)	0.22 (0.02–1.04)	0.19* (0.02–0.91)
Health & social worker	34 (12.4)	4 (30.8)	0.38 (0.01–2.71)	0.43 (0.01–3.10)
Criminal justice agency	49 (17.9)	1 (7.7)	3.12 (0.66–11.96)	3.23 (0.68–12.68)
Mode of onset				
Acute	121 (43.7)	3 (27.3)	0.48 (0.08–2.07)	0.42 (0.07–1.85)
Gradual	56 (20.2)	-	0.26 (0.00–1.63)	0.28 (0.00–1.80)
Insidious	100 (36.1)	8 (72.7)	4.69* (1.10–28.09)	5.17* (1.18–31.51)

*DUP* duration of untreated psychosis, *GP* general practitioner, *IQR* 25th and 75th Percentiles range, *S.D.* standard deviation, *df* degree of freedom, *d* days, *CI* confidence intervals, *FEP-C (control) group* FEP patients who present to mental health services for FEP without prior contact with the prodromal services, *PROD (prodrome) group* FEP patients who had first presented to the prodromal services with the ARMS and who, by definition, subsequently transitioned to FEP

*p < 0.05, ** *p* < 0.01, ***p < 0.001

^a^Adjusted for age at the first contact with mental health services

### Socio-demographic characteristics, DUP and pathways to care: FEP-C vs FEP-P groups

Of all 55 (16.3% of *n* = 338) FEP patients who had prior contact with the prodromal services, 75% (*n* = 41/55) were already experiencing a full psychotic episode at the time of first contact with prodromal services (i.e., FEP-P). This group of patients was younger (mean = 24.7 years, S.D. = 4.4) than those FEP patients who did not come in contact with the prodromal services prior to developing a psychotic disorder (mean = 27.9 years, S.D. = 5.5) (*t*(322) = 3.56, *P* < 0.001). On the first contact with mental health services, 63% (*n* = 26/41) of the FEP-P group lived with members of their family or partners compared to 46% (*n* = 125/274) of the FEP patients without a prior contact with the prodromal services (i.e., FEP-C group) (*x* ^*2*^ = 6.77, *P* = 0.03) (Additional file 1). Further, the pathways to care differed among these two groups: 46% (*n* = 18/39) of the FEP-P group were referred to mental health services by their local GPs, while 45% (*n* = 124/274) of the FEP-C group were referred by the emergency medical services; 17.9% (*n* = 49/274) of the FEP-C group, compared to 7.7% (*n* = 3/39) of the FEP-P group, came in contact with mental health services via the criminal justice agencies. The results of the exact logistic regression analysis highlighted that the FEP-P group was more likely to make the first contact with mental health services via GPs when compared to those patients who did not have prior contact with the prodromal services (Additional file 2).

## Discussion

We found that 4.1% of patients presenting to mental health services with first episode psychosis (FEP) had previously presented to the prodromal services with the ARMS and subsequently transitioned to a psychotic disorder. Although such services are well-known locally, it may be that recognising appropriate cases and directing them to the prodromal service is simply beyond the skills and training of most referrers [37]. The task of effectively detecting the true ARMS cases based on referrals and help-seeking behaviour rather than epidemiological surveys is clearly challenging especially given the lack of sensitive and specific biomarkers indicative of the prodromal phase of psychosis [38, 39]. If these figures are replicated in other settings with similar prodromal services, this will suggest that the promise of early detection of those at high risk for transition to clinical psychosis with the aim of primary prevention, on a large scale, is still some way off. Even if effective, safe, acceptable and efficient interventions were readily available, our findings indicate that we are not yet in a position to apply them in a way which could make anything but a small impact on the incidence of psychosis.

### How could this very low figure be improved?

We found that around 77% of all referrals to the prodromal services were made by health professionals such as local general practitioners (GPs) and other health workers. Clearly, this pathway leaves out young individuals developing psychosis who do not seek help [40, 41] or are not registered with GPs. Similarly, migrants may be less likely to be registered with GPs and may have less trusting attitudes toward mental health professionals [42]. Indeed, in the present study we found that most prodromal patients who came in contact with the prodromal services were born in the UK. Moreover, the likelihood of help-seeking is influenced by the mode of onset of psychotic symptoms [37]. Previous studies showed that patients in less symptomatic states were more likely to seek help from their GPs [37]. Considering that 44% of all our FEP patients had an acute onset of psychotic symptoms, it is not surprising that many would not have sought help via GPs and thus would have not accessed the prodromal services.

The age of first contact was younger in FEP patients who had first presented to the prodromal services and subsequently transitioned to psychotic disorder than in the FEP group without prior contact with prodromal services. Even though these results could be an artefact of the age limit imposed by prodromal services, they may well indicate that the prodromal services are successful in reaching out to younger clients. There was some evidence that DUP differed between the groups, but small samples means this did not reach *P* < 0.05; therefore caution is needed when drawing conclusions. It has been shown that the mode of onset of first psychotic symptoms is one of the strongest determining factors of length of DUP [31]. Indeed, the vast majority of our prodromal group had an insidious mode of onset of their first psychotic symptoms. With slow onset of first symptoms it may be difficult to distinguish the first indicators of the illness from other motivational or developmental difficulties [32, 43]. Further we note that ‘insidious onset’ could also encompass onset with predominantly negative symptoms. However, such symptoms were not specifically rated. Members of their families or close friends may be also less likely to encourage these individuals to seek help when the onset of symptoms is spread over a long period [32]. It is possible of course that treatment delay could have been even longer for these individuals had it not been for the presence of prodromal services. The evidence from the literature suggests that the type of professional with whom the first contact is made following onset of psychosis is an important factor in determining the length of DUP [20, 41, 43]. Considering that GP attendance is associated with a prolonged DUP [41], while the emergency medical services and criminal justice agency are associated with a substantially shorter DUP [20], it may be that those who have an acute onset are likely to access care quickly and, by virtue of being already psychotic, tend to bypass the prodromal services. Given this, prodromal services appear to be the services for slow developing psychosis.

Nonetheless, a recent retrospective study conducted in South London [21] showed that those FEP patients who presented to the prodromal services up to one year before making the transition to a psychotic disorder had a median DUP of 7 days, substantially shorter than the one we observed (median 19 days). However, these differences can be explained by differences in measurement and problems inherent to the DUP construct. The claim that early intervention services reduce DUP relative to generic clinical services [21] is critically dependent on whether the time between the earliest report of symptoms and the beginning of the first treatment under care of early intervention services is taken as the DUP. Alternatively, the beginning of DUP is taken as a ‘reset’ after such an intervention unless or until the individual subsequently develops their first episode of clinical psychosis. One approach that would help avoid this problem is to clearly differentiate between the duration of the prodromal period, defined as the period from the first unspecific symptoms related to psychosis to the first continuous (present most of the time) psychotic symptom [44] and the actual DUP. This method would highlight whether and how much the prodromal services benefit the patients before they make the transition to a FEP and how lasting these benefits are over the subsequent course of illness. Unfortunately, we were unable to examine duration of prodromal period in the present study.

Additionally, we found that around 75% of all patients referred to the prodromal services and who ultimately went psychotic, were already experiencing their first episode psychosis at the time of contact with the prodromal services. Although this supports the notion that the prodromal services are successful in detecting FEP patients who are in turn promptly referred to more appropriate early intervention services, the results of the present study do not suggest that the prodromal services provide additional functions by detecting individuals with incipient first onset of psychosis who otherwise would not have had access to mental health services.

### Methodological considerations

This is the first study to investigate the differences in pathways to care and clinical presentation between heterogeneous groups of FEP patients resident in inner city deprived areas of South East London. We utilized a large sample size of well-characterised incidence cases from both inpatient and outpatient settings, which is representative of actual clinical practice. The SLaM BRC Case Register, which was the primary source of information for the present study, has a near 100% clinical coverage in its boroughs [24]; this further enhanced the generalizability of our findings.

The results of the present study should be interpreted in light of methodological limitations. It may be argued that extracting information from clinical records may not always produce reliable data. For example, for the purposes of determining DUP from clinical records, treating clinicians might not always have recorded in the notes when psychotic symptoms began and their magnitude, thus potentially introducing bias. Although we utilised an operationalised definition of DUP, we did not undertake reliability checks of the information used in this construct. Having said that the distribution of DUP reported in this study is consistent with other research [45–47]. The quality and completeness of information recorded in the electronic notes for each patient inevitably varied and this may have introduced some bias. Further, case register information is limited to people who sought help for their symptoms and thus have accessed services. This however excludes undiagnosed mentally ill individuals within the community. It is also feasible that some of the patients might have sought or purchased mental healthcare elsewhere for a psychotic disorder and thus would not have been registered in the SLaM BRC Case Register, nor included in the present study. The number of patients with FEP who came through the prodromal services was relatively small; therefore, caution is needed when interpreting the results. Finally, the data relating to living circumstances, relationship status and employment provide crude proxies for social networks and as such they can only hint at the potential role of social contexts and networks in influencing the pathway to care.

## Conclusion

Under the current pathways to care, only a small fraction of individuals (4.1%) who present with a FEP to the main secondary mental health provider actually come in contact with the prodromal services before making a subsequent transition to a psychotic disorder. Much of the work of the prodromal services, and by implication similar prodromal programmes, is spent dealing with people who either will never become psychotic or alternatively are already in their FEP. While this signifies an appetite for a variety of flexible services to care for people with early psychosis, it highlights a greater challenge in providing care for people before they develop psychosis and to therefore prevent it or catch it early. Maintaining contact with all such people and responding when interventions are required takes considerable resources. Our findings also imply that research based on the view that at-risk patients recruited through prodromal services capture a process or phase in the illness that is typical of the majority of FEP patients may be questioned.

## Additional files

Additional file 1: Comparisons in socio-demographic characteristics on presentation at first contact with mental health services between FEP-C (control) and FEP-P (psychosis) groups (DOCX 18 kb)

Additional file 2: Comparisons in clinical presentation characteristics and pathways to care between FEP-C (control) and FEP-P (psychosis) groups (DOCX 16 kb)

## Abbreviations

ARMS: At Risk Mental State
BRC: Biomedical Research Centre
CRIS: Case Register Interactive Search tool
DUP: Duration of untreated psychosis
EU-GEI: European Union Gene-Environment Interactions
FEP: First episode of psychosis
GP: General Practitioner
ICD: International Classification of Diseases
IQR: Inter-quartile range
SCAN: Schedules for Clinical Assessment in Neuropsychiatry
SLaM: South London and Maudsley NHS Foundation Trust
WHO: World Health Organisation
