# Supplementary material for: Only a small proportion of patients with first episode psychosis come via prodromal services: a retrospective survey of a large UK mental health programme
Source: BMC Psychiatry. 2017 Aug 25;17:308. doi: 10.1186/s12888-017-1468-y (PMC5574213; doi:10.1186/s12888-017-1468-y)
Supplement: Supplementary file 1 — Comparisons in socio-demographic characteristics on presentation at first contact with mental health services between FEP-C (control) and FEP-P (psychosis) groups (DOCX 18 kb) [file 12888_2017_1468_MOESM1_ESM.docx]

**Additional file 1.** Comparisons in socio-demographic characteristics on presentation at first contact with mental health services between FEP-C (control) and FEP-P (psychosis) groups

| Socio-demographic characteristics | | FEP-C  (n=283; 83.7%) | FEP-P  (n=41; 12.4%) | Statistics | | |
| --- | --- | --- | --- | --- | --- | --- |
|  | | Mean(S.D.)/n(%) | Mean(S.D.)/n(%) | Test statistics | d.f. | *P*-value |
|  | |  |  |  |  |  |
| Age | | 27.9 (5.5) | 24.7 (4.4) | t=3.56 | 322 | <0.001 |
| Range | | 18-37 | 15-35 |  |  |  |
| Gender | |  |  | chi^2^=0.08 | 1 | 0.78^a^ |
|  | Female | 124 (43.8) | 17 (40.5) |  |  |  |
|  | Male | 159 (56.2) | 24 (59.5) |  |  |  |
| Ethnicity | |  |  | chi^2^=0.16 | 2 | 0.92^a^ |
|  | White | 98 (35.1) | 15 (36.6) |  |  |  |
|  | Black | 126 (45.2) | 19 (46.3) |  |  |  |
|  | Other | 55 (19.7) | 7 (17.1) |  |  |  |
| Country of birth | |  |  | chi^2^=3.01 | 1 | 0.08^a^ |
|  | UK | 124 (46.8) | 23 (62.2) |  |  |  |
|  | Not in the UK | 141 (53.2) | 14 (37.8) |  |  |  |
| Education | |  |  | chi^2^=2.85 | 1 | 0.09^a^ |
|  | School | 111 (55.2) | 13 (39.4) |  |  |  |
|  | A-Level or above | 90 (44.8) | 20 (60.6) |  |  |  |
| Employment status | |  |  | chi^2^=0.02 | 1 | 0.88^a^ |
|  | Unemployed | 172 (64.7) | 26 (63.4) |  |  |  |
|  | Employed | 94 (35.3) | 15 (36.6) |  |  |  |
| Marital status | |  |  | chi^2^=0.46 | 1 | 0.56^a^ |
|  | Not in stable relationship | 205 (75.1) | 32 (80.0) |  |  |  |
|  | Married/stable relationship | 68 (24.9) | 8 (20.0) |  |  |  |
| Living arrangements | |  |  | chi^2^=6.77 | 2 | 0.03^b^ |
|  | Alone | 74 (27.0) | 11 (26.8) |  |  |  |
|  | Partner/family | 125 (45.6) | 26 (63.4) |  |  |  |
|  | No stable accommodation | 75 (27.4) | 4 (9.8) |  |  |  |
| Cannabis use | |  |  | chi^2^=0.02 | 1 | 0.90^a^ |
|  | No | 118 (50.2) | 19 (51.3) |  |  |  |
|  | Yes | 117 (49.8) | 18 (48.7) |  |  |  |

S.D, standard deviation; df, degree of freedom; FEP-C (control) group: FEP patients who present to mental health services for FEP without prior contact with the prodromal services; FEP-P (psychosis) group: FEP patients who were found to be already experiencing their FEP at the time of first contact with the ‘prodromal services

^a^ chi-square test

^b^ Fisher's exact test
