# Supplementary material for: Only a small proportion of patients with first episode psychosis come via prodromal services: a retrospective survey of a large UK mental health programme
Source: BMC Psychiatry. 2017 Aug 25;17:308. doi: 10.1186/s12888-017-1468-y (PMC5574213; doi:10.1186/s12888-017-1468-y)
Supplement: Supplementary file 2 — Comparisons in clinical presentation characteristics and pathways to care between FEP-C (control) and FEP-P (psychosis) groups (DOCX 16 kb) [file 12888_2017_1468_MOESM2_ESM.docx]

**Additional file 2.** Comparisons in clinical presentation characteristics and pathways to care between FEP-C (control) and FEP-P (psychosis) groups

| *Clinical presentation and pathways to care* | | FEP-C  (n=283; 83.7%) | FEP-P  (n=41; 12.4%) |  |  |
| --- | --- | --- | --- | --- | --- |
|  | | Mean (S.D.)/n(%) | Mean (S.D.)/n(%) | Unadjusted  OR (95% CI) | Adjusted ^a^  OR (95% CI) |
|  |  |  |  |  |  |
| DUP days Median (IQR) | | 86 (13-368) | 104.5 (52-387) | 1.00 (0.99-1.00) | 1.00 (0.99-1.00) |
|  | |  |  |  |  |
| Source of referral | |  |  |  |  |
|  | General Practitioner | 67 (24.4) | 18 (46.2) | 2.64** (1.25-5.55) | 2.76** (1.28-5.90) |
|  | Emergency services | 124 (45.3) | 12 (30.8) | 0.54 (0.24-1.15) | 0.47 (0.20-1.03) |
|  | Health & social worker | 34 (12.4) | 6 (15.4) | 0.38 (0.07-1.29) | 0.43 (0.08-1.47) |
|  | Criminal justice agency | 49 (17.9 ) | 3 (7.7) | 1.28 (0.41-3.42) | 1.38 (0.43-3.78) |
|  |  |  |  |  |  |
| Mode of onset | |  |  |  |  |
|  | Acute | 121 (43.7) | 15 (36.6) | 0.74 (0.35-1.53) | 0.66 (0.31-1.39) |
|  | Gradual | 56 (20.2) | 12 (29.3) | 1.63 (0.71-3.55) | 1.65 (0.71-3.66) |
|  | Insidious | 100 (36.1) | 14 (34.1) | 0.92 (0.42-1.91) | 1.03 (0.47-2.19) |

DUP, duration of untreated psychosis; IQR, inter-quantile range; df, degree of freedom; FEP-C (control) group: FEP patients who present to mental health services for FEP without prior contact with the prodromal services; FEP-P (psychosis) group: FEP patients who were found to be already experiencing their FEP at the time of first contact with the ‘prodromal services

^a^ Adjusted for age at the first contact with mental health services

**p*<0.05, ** *p*<0.01, ****p*<0.001
